# Changes in lymph node surgery in breast cancer and preoperative drug prescription analysis for postoperative pain management: A retrospective, cross-sectional study

**DOI:** 10.1371/journal.pone.0298270

**Published:** 2024-04-04

**Authors:** Eun Sol Kang, Yu-Cheol Lim, Bo-Hyoung Jang, Yoon Jae Lee, In-Hyuk Ha, Ye-Seul Lee

**Affiliations:** 1 Jaseng Hospital of Korean Medicine, Gangnam-daero, Gangnam-gu, Seoul, Republic of Korea; 2 Jaseng Spine and Joint Research Institute, Jaseng Medical Foundation, Gangnam-daero, Gangnam-gu, Seoul, Republic of Korea; 3 Department of Preventive Medicine, College of Korean Medicine, Kyung Hee University, Kyungheedae-ro, Dongdaemun-gu, Seoul, Republic of Korea; Local Health Authority Caserta: Azienda Sanitaria Locale Caserta, ITALY

## Abstract

This study aimed to investigate the changes in lymph node surgery types and prescription patterns of postoperative medications for pain management in patients with breast cancer using national health insurance claim data from South Korea. The study population comprised patients with at least one record of a principal diagnosis of breast cancer (ICD-10 code: C50) from the national health insurance claim database between 2010 and 2019. Patients who underwent mastectomy or lumpectomy only once were selected for the analysis. Patients who underwent axillary lymph node dissection (ALND) with mastectomy or lumpectomy on the day of surgery were included in the ALND group, whereas those who underwent sentinel lymph node biopsy (SLNB) were included in the SLNB group. Prescription records of opioids before, after and on the date of breast cancer surgery were collected and categorized according to the opioid type. Multivariate logistic regression modeling was used to compare postoperative opioid prescriptions. The proportion of those undergoing ALND among 3,080 patients decreased consistently after 2014, while the proportion undergoing SLNB increased. Although the rate of pain medication prescription on the day of surgery was similar between the two groups, the rate of prescription of postoperative pain medication and anticancer agents was lower in the SLNB group than in the ALND group. Logistic regression modeling showed that the SLNB group had lower odds of receiving opioids than did the ALND group (Odds ratio (OR) = 0.727, Confidence Interval (CI) = 0.546–0.970). A consistent trend was observed when the model was adjusted for neoadjuvant chemotherapy and the use of preoperative pain medications (OR = 0.718, CI = 0.538–0.959). To manage postoperative pain and prevent chronic pain with minimal side effects, sufficient discussion among clinicians, patients, and other healthcare professionals is imperative, along with adequate treatment planning.

## Introduction

Breast cancer is a major cancer in terms of global incidence and mortality. According to the Global Cancer Statistics, 2020 estimates of cancer incidence and mortality, female breast cancer is the most commonly diagnosed cancer, accounting for 11.7% of new cancer cases [1]. The 2018 Korea Central Cancer Registry has reported that breast cancer is the fifth most common cancer in Korea among all cancer types (23,647, 9.7%) and is the most common malignancy among women, accounting for 20.5% of all cancer cases [2].

Breast cancer treatment regimens commonly include chemotherapy, hormone therapy, radiation therapy, and surgical resection, of which surgical management of breast cancer is the foremost option [3]. In the surgical management of breast cancer, axillary lymph node dissection (ALND) traditionally served as a pathological staging and therapeutic tool for patients with breast cancer, playing an essential role in the locoregional management of breast cancer. However, ALND is increasingly associated with complications, such as pain, cutaneous necrosis, collection of lymph fluid, wound infections, numbness or paresthesia, lymphedema, impairment of arm mobility, and shoulder stiffness [4]. In particular, postoperative pain is a major problem affecting the quality of life of patients with breast cancer [5]. According to previous studies, such as those by Schrenk et al. [6] and Peintinger et al. [7], patients who underwent sentinel lymph node biopsy (SLNB) experienced significantly lower postoperative pain than did those who underwent ALND. With the recent development of surgical and diagnostic techniques, SLNB has evolved into a highly useful tool for reducing negative postoperative outcomes and is the preferred choice, where applicable [8]. In addition, several clinical studies have reported that compared to other surgical methods, SLNB allows for the use of a smaller incision, but the oncological outcomes of the technique are not compromised [9–11].

Opioids, which are narcotic analgesics, are an irreplaceable component of pharmacotherapy for pain management; however, because they are highly addictive, their use has become one of the most widely studied national public health problems in the United States over the last 10 years [12, 13]. The use of opioids in South Korea is relatively lower compared with that in other Organization for Economic Co-operation and Development countries [14], and there have been no issues with mortality related to opioid prescriptions in the country to date [15]. However, opioids can be readily prescribed through outpatient visits to primary care physicians and secondary and tertiary hospitals in South Korea [16], and one study using a sample cohort in Korea showed a continuously increasing trend of opioid use from 2002 to 2015 [16]. Accordingly, the awareness of the potential hazards of opioid use is increasing in South Korea.

Most patients with breast cancer undergoing surgery as the mainstay of treatment are routinely discharged with opioid prescriptions for pain management, indicating a high probability of opioid exposure [17]. Previous studies have reported that surgical intervention is associated with the first exposure to opioids in several cases [18] and that the type of surgery for patients with breast cancer is associated with postoperative opioid analgesic prescriptions [19]. Moo et al. [17] reported that most patients undergoing lumpectomy/SLNB could be discharged after surgery with nonsteroidal anti-inflammatory drug/acetaminophen prescriptions alone without resorting to opioid prescriptions for pain control, thereby reducing the number of opioid prescriptions.

To date, there has been a study on differences in narcotic prescriptions as postoperative pain medication with types of surgery, such as mastectomy/lumpectomy [20], but there have been few studies examining differences in the prescription of postoperative pain medications according to the methods of lymph node surgery for patients with breast cancer. Several studies have reported significantly lower postoperative pain in patients who underwent SLNB than in those who underwent ALND [6, 7]; however, whether this is related to the prescription of postoperative pain medications remains unclear.

To address these issues, this study investigated a 10-year trend (2010–2019) in surgical techniques applied for lymph node surgery among patients with breast cancer, focusing on postoperative pain management, and examined whether there was a difference in the prescription of pain medications according to the type of lymph node surgery.

## Materials and methods

### Data collection

In this study, data from January 2010 to December 2019 obtained from the Health Insurance Review and Assessment Service (HIRA)-National Patient Sample (NPS) (provided by the HIRA of South Korea) were used. The HIRA data are insurance claim data generated when a healthcare service provider requests reimbursement from the national health insurance (NHI)for the healthcare service provided. The data provide detailed and diverse information, such as details of the healthcare service provided (treatment, procedure, tests and examinations, and prescription drugs), diagnosis, the amount paid by the payer, patient’s out-of-pocket cost, patient population characteristics, and information on healthcare service providers.

The HIRA-NPS data extraction process is based on a stratified randomized sampling method with stratified age and sex groups of the sampled population, which is a 2% sample (approximately 1,000,000 persons) randomly extracted from the total national population of South Korea (those enrolled in the NHI or Medical Aid). The data are secondary data statistically sampled from raw data, with all personal information and information of the associated corporate bodies being de-identified. It is constructed from information on healthcare services claimed over 1 year from the date of starting medical care in each corresponding year [21, 22].

### Study design and population

Patients who had NHI claims issued at least once with a malignant neoplasm of the breast (ICD-10 code: C50) as the principal diagnosis over the 10-year period from 2010 to 2019 and those who underwent mastectomy or lumpectomy once between February 1 and November 30 in the corresponding year (index period) were included in the analysis. To exclude patients who underwent two or more surgeries in the corresponding year, those who underwent mastectomy, lumpectomy, or lymph node surgery on a date other than the surgery date were excluded from the study patient population.

**Fig 1:**
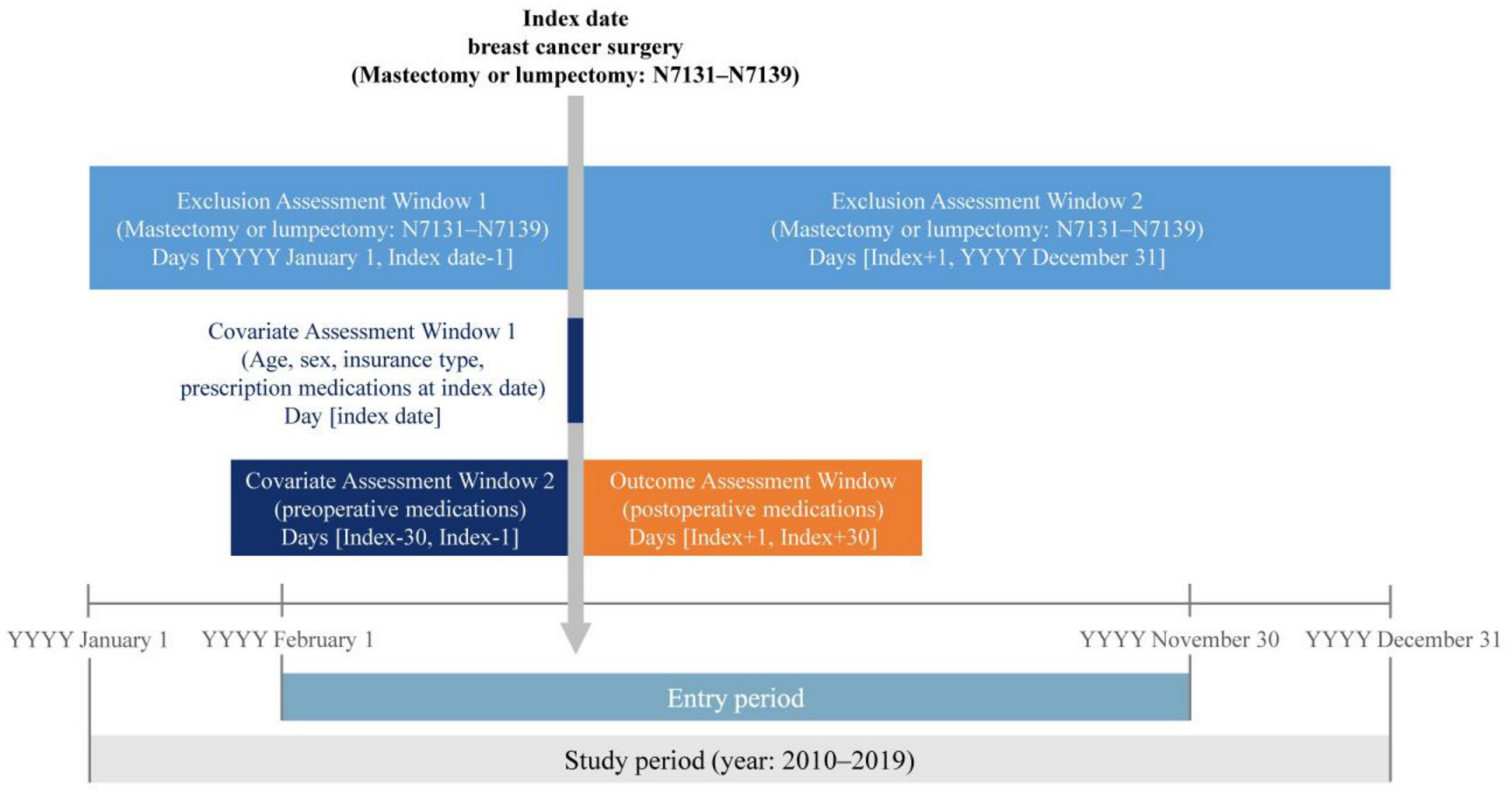
Study design.

The preoperative period was defined as 1 month prior to the surgery, excluding the day of surgery; the index date was defined as the day of surgery; and the postoperative period was defined as 1 month after surgery. Based on these definitions, the medication prescription history of the patients for the preoperative period, index date, and postoperative period was investigated (Fig 1). Patients who underwent ALND along with mastectomy or lumpectomy on the day of surgery were included in the ALND group, whereas those who underwent SLNB along with breast cancer surgery were included in the SLNB group.

### Study outcomes

The baseline characteristics of the patients included age, sex, payer type, type of surgery, and type of preoperative pain medication. Age was categorized into six groups: <30, 30–39, 40–49, 50–59, 60–69, and ≥70 years. Payer type was categorized as NHI or Medicaid, and preoperative pain medication was categorized into four types. The number of patients and percentage of patients based on the type of surgery were estimated for each year during the study period. The pre- and postoperative period medications (medications prescribed during 1 month before and after breast cancer surgery and dispensed from the pharmacy and hospital) and the medications on the index date (day of surgery) were classified into nine categories according to the Anatomical Therapeutic Chemical (ATC) code of the active ingredients of each medication. The frequency of prescriptions for each category was analyzed from 2010 to 2019. Among these, pain medications were further subdivided according to the World Health Organization (WHO) three-step analgesic ladder: non-opioid analgesics for the first step, tramadol for the second step, and opioids for the third step [23]. The pain medications were assigned into categories based on the drug that provided the strongest pain relief among the pain medications prescribed during each period. S1 Table lists the ATC code classifications.

Another outcome was the number of opioid prescriptions during the postoperative period according to the type of surgery. Opioids included morphine, hydromorphone, oxycodone, dihydrocodeine, codeine, hydrocodone, pethidine, fentanyl, pentazocine, buprenorphine, butorphanol, nalbuphine, tapentadol, sufentanil, remifentanil, and tramadol. Although tramadol is generally included in the opioid category, it is not classified as a narcotic analgesic in South Korea; thus, tramadol was separately analyzed.

### Statistical analyses

The number of patients and percentages (%) under the categorical variables of baseline characteristics, ALND and SLNB (type of surgery) cases by year, and medications for different prescription periods were reported. Multivariate logistic regression analysis was performed to calculate the odds ratios (ORs) for opioid prescriptions and 95% confidence intervals (95% CIs). The year of surgery, age, payer type, preoperative period of pain medications for each step based on the WHO analgesic ladder, and status of anticancer agent utilization in the preoperative period were considered covariates in the analysis [24]. Model 1 was adjusted for the year of surgery, and Model 2 was adjusted for age, payer type, and year of surgery. Model 3 was adjusted for preoperative pain medications and preoperative anticancer drugs in addition to the covariates of Model 2. All analyses were performed using the SAS software (version 9.4, SAS Institute, Cary, NC, USA), and a two-sided p-value of <0.05 was considered statistically significant.

### Ethical considerations

This study was exempt from institutional review board (IRB) review by our hospital’s IRB (IRB file no. JASENG 2022-10-008) because we used existing publicly available data, and the patients’ information could not be directly identified through identifiers linked to the patients. Patient consent was not required, as this study used a secondary database with de-identified patient information.

## Results

**Fig 2:**
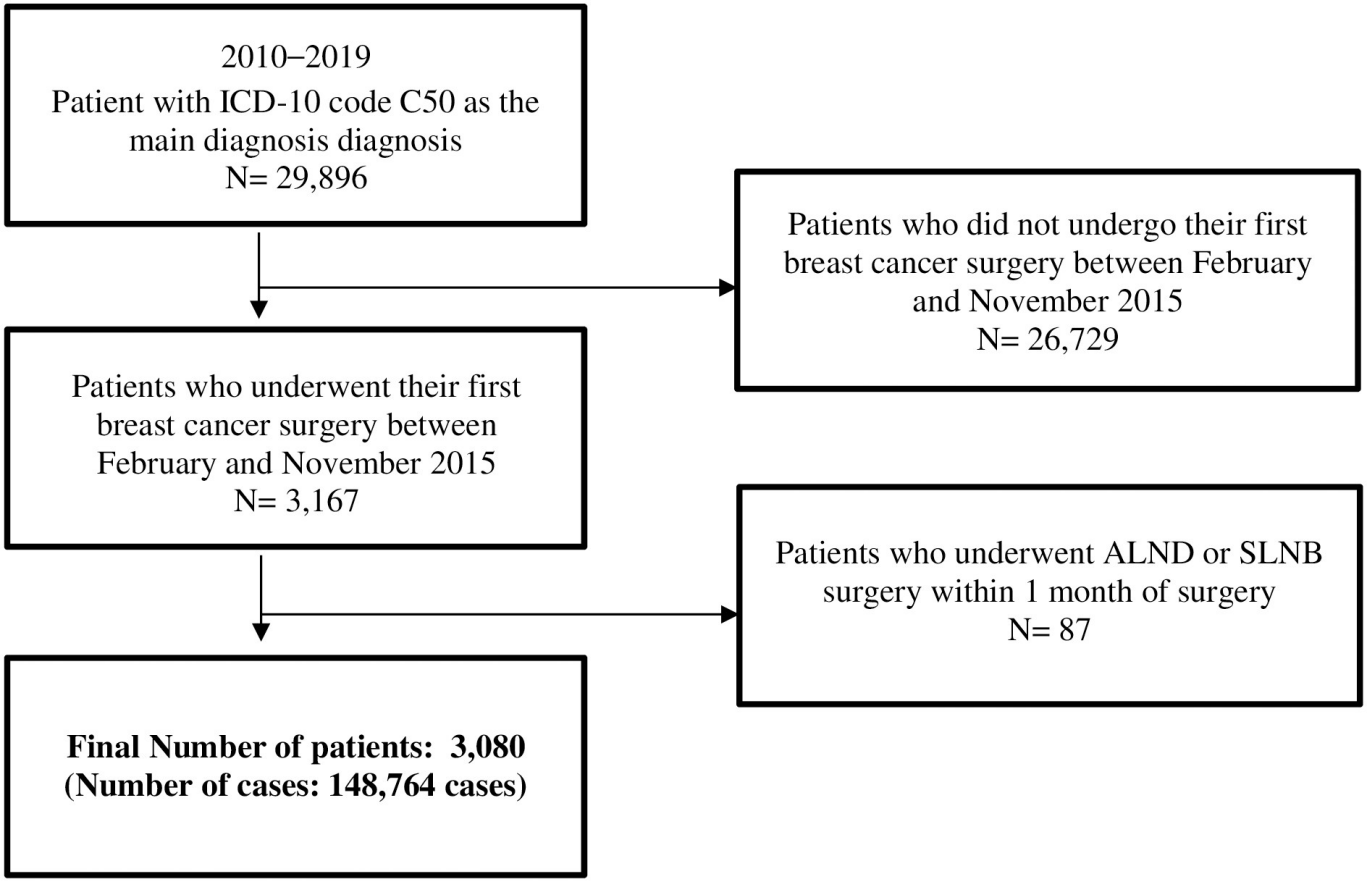
Study patient selection.

A total of 29,896 patients with NHI claim data with malignant neoplasm of the breast (ICD-10 code: C50) as the principal diagnosis at least once during the 10-year period from 2010 to 2019 were identified. Among these patients, those who did not undergo mastectomy or lumpectomy from February 1 to November 30 for each applicable year (n = 26,729) and those who underwent mastectomy, lumpectomy, or lymph node surgery on a day other than the index date (n = 87) were excluded from the analysis. A total of 3,080 patients were included in the final study sample (Fig 2).

Regarding the age group of patients with breast cancer included in this study, patients in their 40s accounted for the highest proportion (n = 1,118, 36.30%), followed by those in their 50s (n = 710, 23.05%) and 30s (n = 673, 21.85%). Among the age groups, patients in their 40s accounted for the highest proportion in both the ALND (n = 672, 37.19%) and SLNB (n = 446, 35.04%) groups, showing a similar distribution to that of the total patient sample. Most patients were women (female, n = 3,068 [99.61%]; male, n = 12 [0.39%]). The NHI was the dominant payer type (n = 2,961, 96.14%), and a small number of patients received Medicaid (n = 119, 3.86%). There were no significant differences between the ALND and SLNB patient groups in terms of age, sex, and type of insurance. The analysis of the prescription records during the preoperative period (1 month prior to the index date) for all breast cancer patients included in this study showed that the majority of patients (n = 2,745, 89.12%) were not prescribed any painkillers. Among those who received prescriptions, non-opioid analgesics were prescribed to 214 patients (6.95%), tramadol to 55 patients (1.79%), and opioids other than tramadol to 66 patients (2.14%). The percentage of patients prescribed preoperative non-opioid analgesics or tramadol was slightly higher in the ALND group than in the SLNB group (non-opioid analgesics: 8.30% vs. 5.03%, tramadol: 2.10% vs. 1.34%) (Table 1).

**Table 1:** Baseline characteristics of the study population.

Category		Total (n = 3,080)		ALND (n = 1,807)		SLNB (n = 1,273)	
		N	Percent	N	Percent	N	Percent
Age	<30	34	1.1	19	1.1	15	1.2
	30–39	334	10.8	220	12.2	114	9.0
	40–49	1,045	33.9	616	34.1	429	33.7
	50–59	906	29.4	540	29.9	366	28.8
	60≤	761	24.7	412	22.8	349	27.4
Sex	Male	12	0.4	7	0.4	5	0.4
	Female	3,068	99.6	1,800	99.6	1,268	99.6
Payer type	NHI	2,961	96.1	1,732	95.9	1,229	96.5
	Medicaid	119	3.9	75	4.2	44	3.5
Preoperative period painkiller	No painkiller	2,745	89.1	1,581	87.5	1,164	91.4
	Step 1 painkiller	214	7.0	150	8.3	64	5.0
	Step 2 painkiller	55	1.8	38	2.1	17	1.3
	Step 3 painkiller	66	2.1	38	2.1	28	2.2

ALND, axillary lymph node dissection; SLNB, sentinel lymph node biopsy; NHI, national health insurance

**Fig 3:**
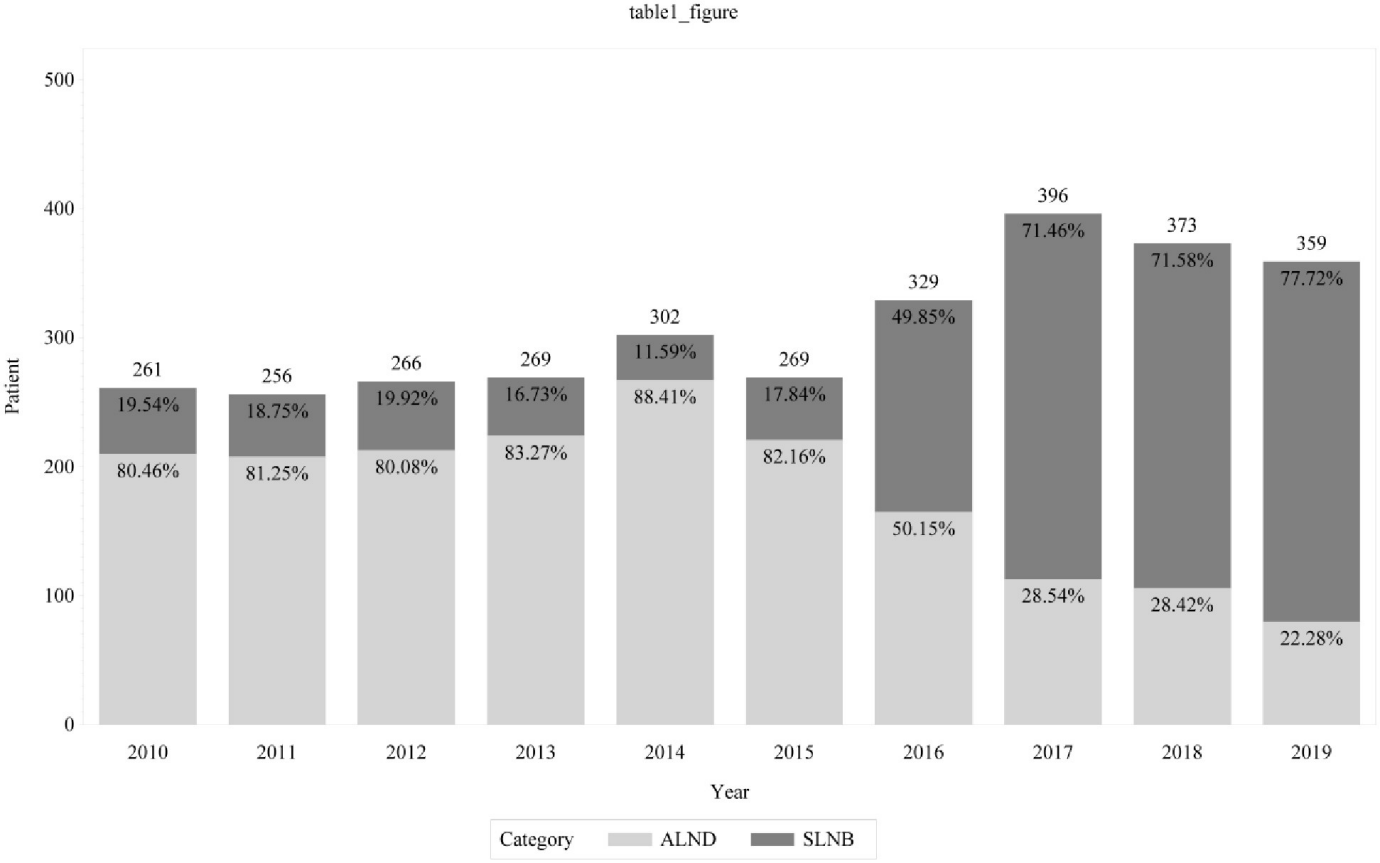
Trends in the types of lymph node surgery from 2010 to 2019. ALND: axillary lymph node dissection; SLNB: sentinel lymph node biopsy.

The examination of the annual trend of changes in the number of patients who underwent lymph node surgery revealed that the total number of patients (irrespective of the type of surgery) increased from 261 in 2010 to 359 in 2019, and the proportion of patients who underwent SLNB increased from 19.5% (n = 51) in 2010 to 77.7% (n = 279) in 2019. In contrast, the number of patients who underwent ALND decreased from 80.5% (n = 210) in 2010 to 22.3% (n = 80) in 2019 (Fig 3).

The prescription rates of pain-related medications and anticancer drugs were analyzed for the ALND/SLNB patient groups by categorizing them into the preoperative period, index date, and postoperative period (Table 2). The numbers of patients prescribed non-opioid analgesics during the postoperative period (for 1 month after surgery) were 362 (20.03%) in the ALND group and 222 (17.44%) in the SLNB group. Tramadol was prescribed during the postoperative period in 122 (6.75%) and 70 (5.50%) patients in the ALND and SLNB groups, respectively. The numbers of patients prescribed opioids were 91 (5.04%) in the ALND group and 48 (3.77%) in the SLNB group. The percentage of patients prescribed postoperative pain medications was higher in the ALND group than in the SLNB group. In contrast, there was no significant difference between the two groups in terms of the percentage of painkiller prescriptions on the index date (non-opioid analgesics: 90.81% vs. 90.34%, tramadol: 34.92% vs. 36.21%, opioids: 94.63% vs. 94.58%). Overall, the percentages of preoperative/index date/postoperative anticancer drug prescriptions were higher in the ALND group than in the SLNB group. The data for the prescription of drugs other than painkillers and anticancer drugs are shown in S2 Table.

**Table 2:** Preoperative drug utilization for pain management.

Drug category	ALND (n = 1,807)						SLNB (n = 1,273)					
	Preoperative period		Index date		Postoperative period		Preoperative period		Index date		Postoperative period	
	No. of patients	Percent	No. of patients	Percent	No. of patients	Percent	No. of patients	Percent	No. of patients	Percent	No. of patients	Percent
Non-opioid analgesics	175	9.7%	1,641	90.8%	362	20.0%	75	5.9%	1,150	90.3%	222	17.4%
Tramadol	46	2.5%	465	25.7%	122	6.8%	23	1.8%	262	20.6%	70	5.5%
Opioids	38	2.1%	1,710	94.6%	91	5.0%	28	2.2%	1,204	94.6%	48	3.8%
Anesthetics (N01AH*)	-	-	1,640	90.8%	9	0.5%	1	0.1%	1,168	91.8%	4	0.3%
Analgesics (N02A*)	38	2.1%	631	34.9%	84	4.6%	27	2.1%	461	36.2%	45	3.5%
Anticancer	253	14.0%	186	10.3%	1,074	59.4%	112	8.8%	77	6.0%	756	59.4%

ALND, axillary lymph node dissection; SLNB, sentinel lymph node biopsy

*Anatomical Therapeutic Chemical classification system

All logistic regression models showed that the prescription of opioids, including tramadol, was significantly lower in the SLNB group than in the ALND group (Model 1: OR 0.727, 95% CI 0.546–0.970; Model 2: OR 0.737, 95% CI 0.553–0.981; Model 3: OR 0.718, 95% CI 0.538–0.959). In contrast, the prescription of opioids other than tramadol was lower in the SLNB group than in the ALND group; however, the difference was not statistically significant (Table 3).

**Table 3:** Association between postoperative opioid prescription and types of breast cancer surgery.

Surgery	Model	Opioids^#^ including tramadol			Opioids^#^		
		Odds ratio	(95% CI)	P-value	Odds ratio	(95% CI)	P-value
SLNB (ref. ALND)	Model 1*	0.727	(0.546–0.970)	0.030	0.764	(0.504–1.158)	0.205
	Model 2^†^	0.737	0.553–0.981	0.037	0.771	(0.510–1.165)	0.217
	Model 3^‡^	0.718	(0.538–0.959)	0.025	0.761	(0.503–1.151)	0.196

*Model 1 adjusted for surgery year

^†^Model 2 adjusted for age, payer type, and surgery year

^‡^Model 3 adjusted for age, payer type, surgery year, preoperative period painkiller use, and preoperative period anticancer drug use

^#^Opioid category includes both anesthetic (N01AH*) and analgesic (N02A*) opioids as per the Anatomical Therapeutic Chemical classification system.

## Discussion

In this study, we investigated the prescription status of postoperative pain medications for patients with breast cancer who underwent lymph node surgery in South Korea from 2010 to 2019 using NHI claim data from the HIRA and examined whether the type of surgery had an effect on the prescription of pain medications. The number of patients who underwent lymph node surgery showed an overall increasing trend over time; however, since 2014, the number of ALND cases has steadily decreased, while the number of SLNB cases has increased. The percentage of pain medication prescriptions on the index date was similar between the two patient groups, but for the prescription of postoperative pain medication and anticancer drugs, the prescription ratio was higher in the ALND patient group than in the SLNB patient group. In addition, the analysis based on logistic regression models to examine the effect of the lymph node surgery method on the prescription of postoperative pain medications demonstrated that the odds of opioid prescription were higher in the ALND group than in the SLNB group in most of the models used in the logistic regression analysis.

Recently, the rate of early detection of breast carcinoma has been increasing owing to the development of breast cancer screening and an increase in the screening rate. Accordingly, the surgical approach to breast cancer shows a trend in the surgical field of minimizing surgery-related morbidity and complications [25, 26]. In support of such trends, several clinical studies have reported that SLNB allows less extensive surgery without affecting oncological outcomes [8]. The publication of results from the American College of Surgeons Oncology Group (ACOSOG) Z0011 randomized clinical trial and dissemination of a regional guideline were associated with a considerable decrease in ALND rates [27]. Furthermore, SENTINA study showed SNLB to be a reliable diagnostic method with high detection rates before neoadjuvant chemotherapy [28], and SN FNAC study reported that the use of sentinel node biopsy after NAC can reduce unnecessary axillary node dissection by more than 30% [29]. The Z1071 study published in 2013 shows a similar result, suggesting SLNB to a reliable alternative to ALND [30]. This study shows a corresponding trend of a continuous increase in SLNB rates since 2014. A prospective observational study using the Korean Breast Cancer Registry reported a steady decrease in the proportion of patients who underwent ALND between 2009 and 2018 (85.2% in 2009 to 47.5% in 2018) [31]. However, in the present study, a rapid change was observed in 2016. This study showed relatively different results, as it observed the trend of surgery occurrence in the entire population, whereas Cha et al. (2022) selected and tracked patients according to the same criteria as that of the Z0011 clinical trial. In comparison to the rapid decrease in ALND frequency observed in the United States and Europe following the publication of the ACOSOG Z0011 study results and guideline revisions, changes in surgical rates among Asian women with breast cancer with different baseline characteristics have been slower, owing to uncertainty regarding the effectiveness of the new strategy.

For patients with stage 1 and 2 breast cancer, postoperative pain management plays an essential role in minimizing the risk of chronic pain, collectively referred to as “persistent postmastectomy pain” [32, 33]. To examine the status of pain management after lymph node surgery in patients with breast cancer, this study analyzed the percentage of prescriptions of pain medication according to surgery type for the preoperative period, index date, and postoperative period. The results showed that the percentage of patients who were prescribed severe pain medications was higher in the ALND group than in the SLNB group. According to a previous study, the prevalence of lymphedema, a postoperative complication, was high in the ALND patient group [34], and patients with lymphedema were prescribed more pain-related medications due to pain [35]. Taken together, recent trends in guidelines [36] and related studies suggest the need for careful attention to pain management for complications such as lymphedema, in addition to direct pain control resulting from surgery, owing to the significantly wider incision range and invasiveness of ALND compared with those of SLNB.

Anticancer therapy has also been reported to be a key factor in pain management in patients with breast cancer. Domestic and international guidelines recommend or approve neoadjuvant chemotherapy for patients with specific clinical conditions [37, 38]. However, according to a previous study by Schott et al., patients receiving neoadjuvant chemotherapy had a high risk of developing peripheral neuropathy, a representative side effect of chemotherapy, and other types of side effects, such as impaired cardiac function, nausea, and vomiting, which may affect surgical outcomes after chemotherapy [39, 40]. Lymph node metastasis is an important factor that influences the choice of surgical treatment. Because the presence of lymph node metastases could not be assessed from the insurance claim data used in this study, we controlled for the effect of lymph node metastases by adjusting for neoadjuvant chemotherapy. Model 3 showed that the trend of postoperative opioid prescriptions was consistent even after adjustment for neoadjuvant chemotherapy, indicating a significant difference in the trend of opioid prescriptions according to the surgical treatment method.

Opioids are an irreplaceable pharmacotherapy for postoperative pain management [41]. However, a recent study reported that the postoperative exposure of opioid-naïve patients to opioids resulted in approximately 10% of patients with cancer undergoing curative-intent surgery for chronic opioid users [42, 43]. Minimizing unnecessary exposure to opioids and implementing optimal pain management through an appropriate surgical method and postoperative pain management are crucial in managing long-term quality of life. South Korea has recently established standards for opioid prescription by presenting the opioid prescription guidelines for 2017 and 2021 [44]. However, no domestic guidelines have been explicitly set for the general use of pain medications, including opioids, for postoperative pain management in patients with breast cancer. The findings of this study are significant in that they demonstrate the status of drug prescriptions for postoperative pain management in this situation.

With the increasing survival rate of patients with breast cancer, improvement in their long-term quality of life and return to normal daily activities are considered the main treatment goals. Therefore, the clinicians’ judgment to balance pain control and minimization of side effects, which have a trade-off relationship, plays a critical role. Healthcare professionals should be careful in selecting pain medications and adjusting doses according to the individual conditions and circumstances of patients and strive to improve the quality of life of patients by achieving maximal pain management with minimal side effects.

### Strengths and limitations

This study analyzed a database that adequately represents the national patient population of South Korea and examined the current status of lymph node surgery types and the prescription of pain medications for postoperative pain management in patients with breast cancer. Additionally, data from 2010 to 2019 were used to analyze relatively recent trends and patterns in healthcare service utilization. In particular, under the current circumstances, in which less-invasive surgical methods are preferred compared to clinical practices in the past, the association between the lymph node surgery method and the prescription of postoperative pain medication was investigated.

Nevertheless, this study has some limitations. First, as this was a cross-sectional study based on NHI claims, only the association, not the causal relationship, between the prescription of postoperative pain medication and the lymph node surgery method could be investigated, and it was not possible to examine the effect of disease severity (staging), clinical conditions of the patients, and extent of the disease. Furthermore, the cross-sectional nature of the database allowed only a limited number of patients who received surgery in the diagnosed year after the breast cancer diagnosis to be included in the study. Second, owing to the nature of HIRA claim data, the medical records of patients can only be viewed and assessed for up to 1 year; therefore, it was difficult to analyze or determine the average duration of drug prescription and changes in prescriptions for each patient in long-term. In addition, because the NPS data were obtained by a stratified sampling of approximately 2% of the Korean population, it may be difficult to generalize the findings of this study to the prescription patterns of all patients with breast cancer in South Korea. Third, the characteristics of prescription drugs were determined by the combination of the number of prescriptions and the number of prescription days, but specific details of prescriptions, such as the frequency of drug utilization, the total number of days of drug administration, and administered dose, could not be included in the analysis. Therefore, defined daily dose (DDD) and prescribed daily dose (PDD) of prescription opioids were not considered outcomes of this study. For more accurate and detailed information on drug utilization, an analysis based on DDD or PDD is required, and this needs to be considered in future studies.

## Conclusions

In this study, the NHI claim data provided by the HIRA were used to analyze the types of lymph node surgery patients with breast cancer underwent and the status of pain medication prescriptions for postoperative pain management. The findings of this study confirms the trend of less invasive interventions on lymph nodes, which led to lower odds of exposure to postoperative opioids. This reflects the importance of managing patients’ long-term quality of life in the treatment of breast cancer and related pain control. In order to minimize postoperative pain and opioid use, close monitoring and observation of the patient’s condition to select the appropriate surgical approach is considered an important step. In addition, careful investigation of the long-term recurrence rate in patients with breast cancer is required, and follow-up studies are required for further examination and analysis of these issues.

## Supporting information

S1 Table Classification of medications. (DOCX)

S2 Table Perioperative drug utilization. (DOCX)

## References

[1] Sung H, Ferlay J, Siegel RL, Laversanne M, Soerjomataram I, Jemal A, et al. Global cancer statistics 2020: GLOBOCAN estimates of incidence and mortality worldwide for 36 cancers in 185 countries. 2021;71(3):209–49. 10.3322/caac.21660 33538338

[2] Kang SY, Lee SB, Kim YS, Kim Z, Kim HY, Kim HJ, et al. Breast cancer statistics in Korea, 2018. 2021;24(2):123. 10.4048/jbc.2021.24.e22 PMC8090800 33913273

[3] Franceschini G, Sanchez AM, Di Leone A, Magno S, Moschella F, Accetta C, et al. New trends in breast cancer surgery: a therapeutic approach increasingly efficacy and respectful of the patient. 2015;36(4):145. 10.11138/gchir/2015.36.4.145 PMC4732583 26712068

[4] Dorval M, Maunsell E, Deschenes L, Brisson J, Masse BJJoCO. Long-term quality of life after breast cancer: comparison of 8-year survivors with population controls. 1998;16(2):487–94. 10.1200/JCO.1998.16.2.487 9469332

[5] Vadivelu N, Schreck M, Lopez J, Kodumudi G, Narayan D JTAS. Article Commentary: Pain after Mastectomy and Breast Reconstruction. 2008;74(4):285–96. 18453290

[6] Schrenk P, Rieger R, Shamiyeh A, Wayand W. JC. Morbidity following sentinel lymph node biopsy versus axillary lymph node dissection for patients with breast carcinoma. 2000;88(3):608–14. 10.1002/(sici)1097-0142(20000201)88:3<608::aid-cncr17>3.0.co;2-k 10649254

[7] Peintinger F, Reitsamer R, Stranzl H, Ralph G. JBjoc. Comparison of quality of life and arm complaints after axillary lymph node dissection vs sentinel lymph node biopsy in breast cancer patients. 2003;89(4):648–52. 10.1038/sj.bjc.6601150 PMC2376906 12915872

[8] Zahoor S, Haji A, Battoo A, Qurieshi M, Mir W, Shah M. Sentinel lymph node biopsy in breast cancer: a clinical review and update. JJobc. 2017;20(3):217–27. doi: 10.4048/jbc.2017.20.3.217 28970846 PMC5620435

[9] Giuliano AE, Ballman KV, McCall L, Beitsch PD, Brennan MB, Kelemen PR, et al. Effect of axillary dissection vs no axillary dissection on 10-year overall survival among women with invasive breast cancer and sentinel node metastasis: the ACOSOG Z0011 (Alliance) randomized clinical trial. 2017;318(10):918–26. 10.1001/jama.2017.11470 PMC5672806 28898379

[10] Galimberti V, Cole BF, Zurrida S, Viale G, Luini A, Veronesi P, et al. Axillary dissection versus no axillary dissection in patients with sentinel-node micrometastases (IBCSG 23–01): a phase 3 randomised controlled trial. 2013;14(4):297–305. 10.1016/S1470-2045(13)70035-4 PMC3935346 23491275

[11] Solá M, Alberro JA, Fraile M, Santesteban P, Ramos M, Fabregas R, et al. Complete axillary lymph node dissection versus clinical follow-up in breast cancer patients with sentinel node micrometastasis: final results from the multicenter clinical trial AATRM 048/13/2000. 2013;20(1):120–7. 10.1245/s10434-012-2569-y 22956062

[12] Cho NR, Chang YJ, Lee D, Kim JR, Ko DS, Choi JJ. JPo. Trends in opioid prescribing practices in South Korea, 2009–2019: Are we safe from an opioid epidemic? 2021;16(5):e0250972. 10.1371/journal.pone.0250972 PMC8115784 33979378

[13] Shah A, Hayes CJ, Martin BCJM. Characteristics of initial prescription episodes and likelihood of long-term opioid use—United States, 2006–2015. Report MW. 2017;66(10):265. 10.15585/mmwr.mm6610a1 PMC5657867 28301454

[14] Studies OJOHP. Addressing problematic opioid use in OECD countries. 2019.

[15] Lee J-HJ. The opioid epidemic and crisis in US: how about Korea? TKJoP. 2019;32(4):243–4. doi: 10.3344/kjp.2019.32.4.243 31569915 PMC6813894

[16] Oh TK, Jeon Y-T. Trends in chronic opioid use and association with five-year survival in South Korea: a population-based cohort study. Choi JWJBJoA. 2019;123(5):655–63. doi: 10.1016/j.bja.2019.08.012 31558315

[17] Moo T-A, Pawloski KR, Sevilimedu V, Charyn J, Simon BA, Sclafani LM, et al. Changing the default: a prospective study of reducing discharge opioid prescription after lumpectomy and sentinel node biopsy. 2020;27(12):4637–42. 10.1245/s10434-020-08886-9 PMC7554186 32734370

[18] Rao R, Jackson RS, Rosen B, Brenin D, Cornett W, Fayanju OM, et al. Pain control in breast surgery: survey of current practice and recommendations for optimizing management—American Society of Breast Surgeons Opioid/Pain Control Workgroup. 2020;27(4):985–90. 10.1245/s10434-020-08197-z PMC7241428 31965373

[19] Park KU, Kyrish K, Terrell J, Yi M, Caudle AS, Hunt KK, et al. Surgeon perception versus reality: opioid use after breast cancer surgery. 2019;119(7):909–15. 10.1002/jso.25395 30737785

[20] Fan B, Valente SA, Shilad S, Al-Hilli Z, Radford DM, Tu C, et al. Reducing narcotic prescriptions in breast surgery: a prospective analysis. 2019;26(10):3109–14. 10.1245/s10434-019-07542-1 31342372

[21] Ryu JG, Kim SY, Park S, Je NK. Current Prescription Status of Contraindicated Drug Combinations Causing Serotonin Syndrome: Analysis of HIRA-NPS Data. Korean Journal of Clinical Pharmacy. 2022;32(4):313–20.

[22] Kim L, Kim JA, Kim S. A guide for the utilization of Health Insurance Review and Assessment Service National Patient Samples. Epidemiol Health. 2014;36:e2014008. Epub 2014/08/01. doi: 10.4178/epih/e2014008 . 25078381 PMC4151963

[23] Anekar AA, Cascella M. WHO analgesic ladder. StatPearls [Internet]: StatPearls Publishing; 2021. 32119322

[24] Shen C, Thornton JD, Gu D, Dodge D, Zhou S, He W, et al. Prolonged Opioid Use After Surgery for Early‐Stage Breast Cancer. The Oncologist. 2020;25(10):e1574–e82. doi: 10.1634/theoncologist.2019-0868 32390251 PMC7543235

[25] Rietman JS, Dijkstra PU, Geertzen JH, Baas P, De Vries J, Dolsma W, et al. Short‐term morbidity of the upper limb after sentinel lymph node biopsy or axillary lymph node dissection for Stage I or II breast carcinoma. 2003;98(4):690–6. 12910511 10.1002/cncr.11545

[26] Luo Y, Zhao C, Gao Y, Xiao M, Li W, Zhang J, et al. Predicting axillary lymph node status with a nomogram based on breast lesion ultrasound features: performance in n1 breast cancer patients. 2020;10:581321. 10.3389/fonc.2020.581321 PMC7653095 33194714

[27] Tsao MW, Cornacchi SD, Hodgson N, Simunovic M, Thabane L, Cheng J, et al. A population-based study of the effects of a regional guideline for completion axillary lymph node dissection on axillary surgery in patients with breast cancer. 2016;23(10):3354–64. 10.1245/s10434-016-5310-4 27342830

[28] Kuehn T, Bauerfeind I, Fehm T, Fleige B, Hausschild M, Helms G, et al. Sentinel-lymph-node biopsy in patients with breast cancer before and after neoadjuvant chemotherapy (SENTINA): a prospective, multicentre cohort study. Lancet Oncol. 2013;14(7):609–18. Epub 20130515. doi: 10.1016/S1470-2045(13)70166-9 . 23683750

[29] Boileau JF, Poirier B, Basik M, Holloway CM, Gaboury L, Sideris L, et al. Sentinel node biopsy after neoadjuvant chemotherapy in biopsy-proven node-positive breast cancer: the SN FNAC study. J Clin Oncol. 2015;33(3):258–64. Epub 20141201. doi: 10.1200/JCO.2014.55.7827 . 25452445

[30] Boughey JC, Suman VJ, Mittendorf EA, Ahrendt GM, Wilke LG, Taback B, et al. Sentinel lymph node surgery after neoadjuvant chemotherapy in patients with node-positive breast cancer: the ACOSOG Z1071 (Alliance) clinical trial. JAMA. 2013;310(14):1455–61. doi: 10.1001/jama.2013.278932 . 24101169 PMC4075763

[31] Cha C, Kim EY, Kim SY, Ryu JM, Park MH, Lee S, et al. Impact of the ACOSOG Z0011 trial on surgical practice in Asian patients: trends in axillary surgery for breast cancer from a Korean Breast Cancer Registry analysis. World Journal of Surgical Oncology. 2022;20(1):198. doi: 10.1186/s12957-022-02673-4 35698188 PMC9195282

[32] Gong Y, Tan Q, Qin Q, Wei C. Prevalence of postmastectomy pain syndrome and associated risk factors: A large single-institution cohort study. JM. 2020;99(20). doi: 10.1097/MD.0000000000019834 32443289 PMC7253604

[33] Beederman M, Bank J. Post-Breast Surgery Pain Syndrome: Shifting a Surgical Paradigm. JP Open RSG. 2021;9(7). 10.1097/GOX.0000000000003720 PMC8301281 34316427

[34] McLaughlin SA, Wright MJ, Morris KT, Sampson MR, Brockway JP, Hurley KE, et al. Prevalence of lymphedema in women with breast cancer 5 years after sentinel lymph node biopsy or axillary dissection: patient perceptions and precautionary behaviors. Journal of clinical oncology. 2008;26(32):5220. doi: 10.1200/JCO.2008.16.3766 18838708 PMC2652092

[35] Lee J, Park IB, Seo HJ. Pain-related Prescribing Patterns and Associated Factor in Breast Cancer Patients. Korean Journal of Clinical Pharmacy. 2021;31(2):115–24. doi: 10.24304/kjcp2021.31.2.115

[36] Lyman GH, Somerfield MR, Bosserman LD, Perkins CL, Weaver DL, Giuliano AE. Sentinel lymph node biopsy for patients with early-stage breast cancer: American Society of Clinical Oncology clinical practice guideline update. J Clin Oncol. 2017;35(5):561–4. doi: 10.1200/JCO.2016.71.0947 27937089

[37] Hamdy O. Neoadjuvant therapy should be the standard of care for every node positive breast cancer patient. JJoBC. 2019;22(1):149–52. doi: 10.4048/jbc.2019.22.e3 30941242 PMC6438838

[38] Korea S. Health insurance review & assessment service, national interest disease statistics of 2017. Retrieved March 13, 2018. 2017.

[39] Schott AF, Hayes DF. Defining the benefits of neoadjuvant chemotherapy for breast cancer. JJocoojotASoCO. 2012;30(15):1747–9. doi: 10.1200/JCO.2011.41.3161 22508810

[40] Lee K. Longitudinal association of resilience with cancer-related symptoms, and quality of life in breast cancer patients undergoing chemotherapy before surgery: master’s thesis]. Seoul: Korea Univ; 2017.

[41] Kim KMJA. Opioid pharmacogenetics in anesthesia and pain management. Medicine P. 2015;10(2):65–76.

[42] Cody Iii HS. Current surgical management of breast cancer. Gynecology JCOiO. 2002;14(1):45–52. doi: 10.1097/00001703-200202000-00008 11801876

[43] Marcusa DP, Mann RA, Cron DC, Fillinger BR, Rzepecki AK, Kozlow JH, et al. Prescription opioid use among opioid-naive women undergoing immediate breast reconstruction. 2017;140(6):1081–90. 10.1097/PRS.0000000000003832 29176408

[44] IM J, Cho JY. Lessons from Using Opioid to the Treatment of Chronic Pain: Focus on Experiences with the Organization for Economic Cooperation and Development Countries. JHP Management. 2021;31(4):409–22.

