# Supplementary material for: Changes in lymph node surgery in breast cancer and preoperative drug prescription analysis for postoperative pain management: A retrospective, cross-sectional study
Source: PLoS One. 2024 Apr 4;19(4):e0298270. doi: 10.1371/journal.pone.0298270 (PMC10994348; doi:10.1371/journal.pone.0298270)
Supplement: S1 Table — (DOCX) [file pone.0298270.s001.docx]

| S1 Table. Classification of medications. | |
| --- | --- |
| Category | ATC code |
| Non-opioid analgesics | M01 (M01AB, M01AC, M01AE, M01AG, M01AH, M01AX), M02AA, M02AB, N02BA, N02BE |
| Tramadol | N02AJ13, N02AJ14, N02AJ15, N02AJ16 |
| Opioids | N01AH, N02AA, N02AB, N02AE, N02AF, N02AJ01, N02AJ02, N02AJ03, N02AJ06, N02AJ07, N02AJ08, N02AJ09, N02AJ17, N02AJ18, N02AJ19 |
| Anticancer drugs | G03XC, L01 (L01AA, L01BA, L01BC, L01CA, L01CD, L01DB, L01EF, L01EH, L01FD, L01XA, L01XC), L02 (L02AB, L02AE, L02BA, L02BG), L04AA, L04AD |
| Hormonal drugs | G03CA, G03GA, H01 (H01AA, H01BA, H01BB), H02AB, H05BA |
| Neuropsychiatric drugs | N02CC, N03 (N03AB, N03AE, N03AF, N03AG, N03AX), N04 (N04AA, N04AC, N04BA, N04BC, N04BD), N05 (N05AA, N05AD, N05AH, N05AX, N05BA, N05BB, N05BE, N05CD, N05CF), N06 (N06AA, N06AB, N06AX, N06BX, N06DA, N06DX), N07 (N07AA, N07AB, N07AX, N07CA, N07XX) |
| Gastrointestinal drugs | A02 (A02AA, A02AB, A02AC, A02AD, A02BA, A02BB, A02BC, A02BX, A02X), A03 (A03A, A03AA, A03AB, A03AC, A03AD, A03AX, A03BA, A03BB, A03F, A03FA), A04 (A04AA, A04AD), A05 (A05A, A05AA, A05AX, A05BA), A06 (A06AB, A06AC, A06AD, A06AG), A07 (A07AA, A07BC, A07CA, A07DA, A07FA), A09AA, A16AX |
| Surgery-related drugs | B01 (B01AA, B01AB, B01AC, B01AD, B01AF, B01AX), B02 (B02AA, B02AB, B02BA, B02BB, B02BC, B02BD, B02BX), G01 (G01AA, G01AF, G01AX), N01 (N01AB, N01AF, N01AX, N01BB) |
| Blood substitutes and nutritional fluids | B05 (B05AA, B05BA, B05BB, B05BC, B05XA, B05XB, B05XC, B05Z, B05ZA) |
| Antimicrobials | J01 (J01AA, J01CA, J01CE, J01CR, J01DB, J01DC, J01DD, J01DE, J01DF, J01DH, J01EE, J01FA, J01FF, J01GB, J01MA, J01XA, J01XD), J02AC, J04 (J04AB, J04AC, J04AK), J05 (J05AB, J05AF, J05AH, J05AP), J06BA, P01 (P01AB, P01BA) |
| Diagnostics | V04 (V04CA, V04CF, V04CH, V04CX), V08 (V08AA, V08AB, V08B, V08BA, V08CA), V09 (V09AB, V09B, V09BA, V09CA, V09DB, V09FX, V09GA, V09GX, V09IX) |
| Lipid-modifying drugs | C10 (C10AA, C10AB, C10AX, C10BA, C10BX) |
| Antihypertensive drugs | C01 (C01AA, C01BD, C01CA, C01DA, C01DX, C01EA, C01EB), C02 (C02CA, C02DB, C02DC), C03 (C03AA, C03BA, C03CA, C03DA, C03DB), C04 (C04AE, C04AF, C04AX), C05 (C05AD, C05BX, C05CA, C05CX), C07 (C07AA, C07AB, C07AG), C08 (C08CA, C08DA, C08DB), C09 (C09AA, C09BB, C09CA, C09DA, C09DB) |
| Diabetes mellitus drugs | A10 (A10AB, A10AD, A10AE, A10BA, A10BB, A10BD, A10BF, A10BG, A10BH, A10BJ, A10BK, A10BX) |
| Others | A01 (A01AC, A01AD), A11 (A11BA, A11CC, A11DA, A11EA, A11EB, A11GA, A11HA), A12 (A12AA, A12AX, A12BA, A12CC), B03 (B03AA, B03AB, B03AC, B03AD, B03BA, B03BB, B03XA), B05CA, B05CB, B05CX, D01 (D01AC, D01AE), D02AE, D06 (D06AX, D06BA, D06BB, D06BX), D07 (D07AA, D07AB, D07AC, D07AD, D07CC), D08 (D08AC, D08AG), D11 (D11AH, D11AX), G02AB, G02CA, G02CB, G04 (G04BD, G04BX, G04CA), H03 (H03AA, H03BA, H03BB), L03 (L03AA, L03AX), M03 (M03AB, M03AC, M03BA, M03BX), M04AA, M05 (M05BA, M05BB, M05BX), M09 (M09AB, M09AX), R01 (R01AD, R01BA), R02 (R02AA, R02AD), R03AA, R03AC, R03AK, R03AL, R03BA, R03BB, R03CA, R03CC, R03DA, R03DC, R05, R05CA, R05CB, R05DA, R05DB, R05FA, R06AA, R06AB, R06AD, R06AE, R06AX, R07AB, R07AX, S01AA, S01AD, S01AE, S01BA, S01CA, S01FA, S01GX, S01JA, S01L, S01XA, S02AA, S02CA, V03AB, V03AE, V03AF, V06DB, V06DX, V07AB |

| **S2 Table. Perioperative drug utilization.** | | | | | | | | | | | | | |
| --- | --- | --- | --- | --- | --- | --- | --- | --- | --- | --- | --- | --- | --- |
| **Drug category** | **ALND** | | | | | |  | **SLNB** | | | | | |
|  | Preoperative period | | Index date | | Postoperative period | |  | Preoperative period | | Index date | | Postoperative period | |
|  | No. of patients | Percent | No. of patients | Percent | No. of patients | Percent |  | No. of patients | Percent | No. of patients | Percent | No. of patients | Percent |
| Antimicrobials | 137 | 7.58% | 1,786 | 98.84% | 369 | 20.42% |  | 69 | 5.42% | 1,238 | 97.25% | 172 | 13.51% |
| Gastrointestinal drugs | 363 | 20.09% | 1,806 | 99.94% | 853 | 47.21% |  | 144 | 11.31% | 1,258 | 98.82% | 439 | 34.49% |
| Blood substitutes and nutritional fluids | 353 | 19.54% | 1,806 | 99.94% | 781 | 43.22% |  | 175 | 13.75% | 1,256 | 98.66% | 372 | 29.22% |
| Diagnostics | 1,312 | 72.61% | 1,233 | 68.23% | 225 | 12.45% |  | 938 | 73.68% | 980 | 76.98% | 126 | 9.90% |
| Hormonal drugs | 228 | 12.62% | 296 | 16.38% | 574 | 31.77% |  | 97 | 7.62% | 152 | 11.94% | 272 | 21.37% |
| Diabetes mellitus drugs | 8 | 0.44% | 92 | 5.09% | 14 | 0.77% |  | 5 | 0.39% | 60 | 4.71% | 5 | 0.39% |
| Lipid-modifying drugs | 9 | 0.50% | 51 | 2.82% | 13 | 0.72% |  | 4 | 0.31% | 49 | 3.85% | 9 | 0.71% |
| Antihypertensive drugs | 128 | 7.08% | 874 | 48.37% | 148 | 8.19% |  | 58 | 4.56% | 662 | 52.00% | 103 | 8.09% |
| Neuropsychiatric drugs | 88 | 4.87% | 1,695 | 93.80% | 363 | 20.09% |  | 45 | 3.53% | 1,124 | 88.30% | 157 | 12.33% |
| Surgery-related drugs | 128 | 7.08% | 1,740 | 96.29% | 207 | 11.46% |  | 103 | 8.09% | 1,241 | 97.49% | 114 | 8.96% |
| Gas | 4 | 0.22% | 1,803 | 99.78% | 8 | 0.44% |  | - | - | 1,232 | 96.78% | 7 | 0.55% |
| Others | 283 | 15.66% | 1,782 | 98.62% | 744 | 41.17% |  | 168 | 13.20% | 1,238 | 97.25% | 482 | 37.86% |
| ALND, axillary lymph node dissection; SLNB, sentinel lymph node biopsy  ^*^Anatomical Therapeutic Chemical classification system | | | | | | | | | | | | | |
