# Supplementary material for: Changes in lymph node surgery in breast cancer and preoperative drug prescription analysis for postoperative pain management: A retrospective, cross-sectional study
Source: PLoS One. 2024 Apr 4;19(4):e0298270. doi: 10.1371/journal.pone.0298270 (PMC10994348; doi:10.1371/journal.pone.0298270)
Supplement: S2 Table — (DOCX) [file pone.0298270.s002.docx]

| **S2 Table. Perioperative drug utilization.** | | | | | | | | | | | | | |
| --- | --- | --- | --- | --- | --- | --- | --- | --- | --- | --- | --- | --- | --- |
| **Drug category** | **ALND** | | | | | |  | **SLNB** | | | | | |
|  | Preoperative period | | Index date | | Postoperative period | |  | Preoperative period | | Index date | | Postoperative period | |
|  | No. of patients | Percent | No. of patients | Percent | No. of patients | Percent |  | No. of patients | Percent | No. of patients | Percent | No. of patients | Percent |
| Antimicrobials | 137 | 7.58% | 1,786 | 98.84% | 369 | 20.42% |  | 69 | 5.42% | 1,238 | 97.25% | 172 | 13.51% |
| Gastrointestinal drugs | 363 | 20.09% | 1,806 | 99.94% | 853 | 47.21% |  | 144 | 11.31% | 1,258 | 98.82% | 439 | 34.49% |
| Blood substitutes and nutritional fluids | 353 | 19.54% | 1,806 | 99.94% | 781 | 43.22% |  | 175 | 13.75% | 1,256 | 98.66% | 372 | 29.22% |
| Diagnostics | 1,312 | 72.61% | 1,233 | 68.23% | 225 | 12.45% |  | 938 | 73.68% | 980 | 76.98% | 126 | 9.90% |
| Hormonal drugs | 228 | 12.62% | 296 | 16.38% | 574 | 31.77% |  | 97 | 7.62% | 152 | 11.94% | 272 | 21.37% |
| Diabetes mellitus drugs | 8 | 0.44% | 92 | 5.09% | 14 | 0.77% |  | 5 | 0.39% | 60 | 4.71% | 5 | 0.39% |
| Lipid-modifying drugs | 9 | 0.50% | 51 | 2.82% | 13 | 0.72% |  | 4 | 0.31% | 49 | 3.85% | 9 | 0.71% |
| Antihypertensive drugs | 128 | 7.08% | 874 | 48.37% | 148 | 8.19% |  | 58 | 4.56% | 662 | 52.00% | 103 | 8.09% |
| Neuropsychiatric drugs | 88 | 4.87% | 1,695 | 93.80% | 363 | 20.09% |  | 45 | 3.53% | 1,124 | 88.30% | 157 | 12.33% |
| Surgery-related drugs | 128 | 7.08% | 1,740 | 96.29% | 207 | 11.46% |  | 103 | 8.09% | 1,241 | 97.49% | 114 | 8.96% |
| Gas | 4 | 0.22% | 1,803 | 99.78% | 8 | 0.44% |  | - | - | 1,232 | 96.78% | 7 | 0.55% |
| Others | 283 | 15.66% | 1,782 | 98.62% | 744 | 41.17% |  | 168 | 13.20% | 1,238 | 97.25% | 482 | 37.86% |
| ALND, axillary lymph node dissection; SLNB, sentinel lymph node biopsy  ^*^Anatomical Therapeutic Chemical classification system | | | | | | | | | | | | | |
